# The Generalized Adaptation Account of Autism

**DOI:** 10.3389/fnins.2020.534218

**Published:** 2020-10-06

**Authors:** Clara Gernert, Peter Falkai, Christine M. Falter-Wagner

**Affiliations:** ^1^Department of Psychiatry, Medical Faculty, LMU Munich, Munich, Germany; ^2^Department of Psychology, University of Cologne, Cologne, Germany

**Keywords:** autism, adaptation, multicausal pathogenesis, connectivity, circadian rhythm, tryptophan, environment, stress

## Abstract

The heterogeneous phenomenology of autism together with diverse patterns of comorbidities led in the past to formulation of manifold theories and hypotheses on different explanatory levels. We scrutinize most recent findings from genetics, neurobiology and physiology and derive testable hypotheses about possible physiological links between domains. With focus on altered sensory perception and neuronal processing in ASD, we assume two intertwined regulatory feedback circuits under the umbrella of genetics and environmental factors. Both regulatory circuits are highly variable between individuals in line with the heterogeneous spectrum of ASD. The circuits set off from altered pathways and connectivity in ASD, fueling HPA-axis activity and distress. In the first circuit altered tryptophan metabolism leads to higher neurotoxic substances and reinforces the excitation:inhibition imbalance in the brain. The second circuit focuses on the impact and interaction with the environment and its rhythms in ASD. With lower melatonin levels, as the pacemaker molecule of the circadian system, we assume misalignment to outer and inner states corroborated from the known comorbidities in ASD. Alterations of the microbiome composition in ASD are supposed to act as a regulatory linking factor for both circuits. Overall, we assume that altered internal balance on cellular and neurophysiological levels is one of the main reasons leading to a lower ability in ASD to adapt to the environment and own internal changing states, leading to the conceptualization of autism as a condition of generalized imbalance in adaptation. This comprehensive framework opens up new perspectives on possible intervention and prevention strategies.

## Introduction

The challenge of autism research to comprehensively unify the array of symptoms in social interaction and communication as well as repetitive and restricted interests and behaviors (American Psychiatric Association [APA], 2013) is unmet. Moreover, autism spectrum disorders (ASD) are characterized by extreme phenomenological heterogeneity. Genetic research in the past decades has shown large concordance rates (Folstein and Rutter, 1977), while the exact genetic mechanisms causing ASD remain elusive, with over 170 candidate genes associated with ASD known to date (SFARI Gene, 2019). Meanwhile, cognitive, neurobiological, endocrinological and environmental theories have been formulated, with each respective level furthering our understanding of ASD but not being able to explain the etiology of symptoms on other levels. As a consequence, giving up on a single explanation of autism has been suggested (Happé et al., 2006). A multicausal pathogenesis converging to the spectrum of autistic phenomenology seems likely. Nevertheless, we believe that a theoretical framework of ASD attempting to unify most recent state-of-the-art findings from diverse levels of explanation can create a synergistic understanding of ASD in its whole complexity.

At this moment, several new leads are being followed in autism research that renew our thinking about neuronal connectivity in ASD (Tomasi and Volkow, 2019), gene x environment interactions (Rossignol and Frye, 2012) and involvement of the gut microbiome (Sarkar et al., 2018; Xu et al., 2019) opening many new questions, in particular on the links between discussed domains.

Thus, here we review the state-of-the-art knowledge in several current key domains of autism research, spanning genetic signaling pathways of neurodevelopment, neuronal connectivity and thalamic filter mechanisms, circadian rhythms, immunology, social functioning, neuroendocrinology, and the gut-brain-axis. We propose viable links between the key domains, generate targeted hypotheses and put forward a comprehensive framework of ASD that allows for the graded phenomenological expression observed across the spectrum.

## Main Article

### Alterations of Neurodevelopmental Signaling Pathways in ASD

Incontestably, ASD is highly heritable and based on a complex genetic etiology. In the latest GWAS the polygenic heterogeneity of autism-subtypes is confirmed qualitatively and quantitatively (Grove et al., 2019). *De novo* mutations, especially copy number variants (CNVs) and gene disrupting point mutations, which are supposed to have a larger effect in ASD, contribute to the individual liability, <5% (Gaugler et al., 2014; Iossifov et al., 2014), far less compared to the overall heritability. Special emphasis should be placed on the recent identification of five risk loci for ASD and seven additional loci that are shared with other traits (Grove et al., 2019).

With a view to converging pathways, genes of the WNT signaling pathway (Kalkman, 2012; Mulligan and Cheyette, 2016; Kumar et al., 2019) as well as calcium signaling and the MAPK signaling pathway are widely associated with ASD (Wen et al., 2016). KCNN2, as a voltage independent Calcium-activated potassium channel, represents a highly significant locus in the genetics of ASD (Grove et al., 2019). Activation of KCNN2 modulates neuronal excitability by membrane hyperpolarization, potentially boosting the risk of an altered excitation/inhibition ratio between neurons. Thus, these genetic alterations likely have a negative effect on intracellular and intercellular communication leading to altered connectivity via synaptic plasticity.

The WNT signaling pathway helps coordinating neurodevelopmental processes like cell proliferation, synaptogenesis, polarity and differentiation (MacDonald et al., 2009). WNT3 as one of the 19 ligands of the WNT signal cascade has been reported to be elevated in the prefrontal cortex of ASD patients (Chow et al., 2012). WNT2, as another ligand, is important for cortical dendrite growth and dendritic spine formation, while alterations of dendritic spines result in neurodevelopmental diseases (Oliva et al., 2013). Prostaglandin E2 as an inflammatory molecule is known to strengthen the canonical WNT-pathway (Wong et al., 2014).

The specific genetic architecture of ASD is still unknown. The interconnection of rare *de novo* mutations and inherited variants of different genes in aspects of transcription and protein networks in ASD, might result in abnormal concentrations of neuroligins, altered interconnection and synapse formation, dysregulation of the excitation/inhibition ratio as well as impairments of the immune system, referring to immune cell activation by Calcium as a core molecule. Moreover, we assume that the heterogeneous spectrum of ASD is amongst others caused by an underlying gradual effect of genetic alterations, while their dysregulation gets reinforced by a proinflammatory profile leading to a vicious circle.

As a major effect of these negative feedback mechanisms, we propose individuals with ASD to suffer from a reduced capacity to physiologically adapt to inner and outer states leading to a dysfunctional homeostasis. This imbalance is affecting the whole organism, as will be spelled out in detail for each building block in the following sections. ASD is thus proposed to be understood as a condition of generalized imbalance in adaptation.

### Connectivity in ASD

Hypothesis 1: Local thalamic underconnectivity and long-range overconnectivity leads to chronic distress.

In several resting-state functional magnetic resonance imaging (rfMRI) studies in the last years, mostly all based on a relatively small sample size, heterogeneous results were found with respect to local and long-range connectivity in ASD that lead to the hypothesis that ASD might present with more local and less long-range connectivity compared to non-autistic people (Belmonte, 2004; Anderson et al., 2011). Results were equivocal though and several rfMRI studies demonstrated long-range overconnectivity between brain regions (Monk et al., 2009; Di Martino et al., 2014; Cerliani et al., 2015). In a recently published study a large number of rfMRI datasets of individuals with ASD (*n* = 565) were compared with datasets of unaffected healthy controls (HC; *n* = 605) using functional connectivity density mapping (Tomasi and Volkow, 2019). The anterior thalamus showed *local underconnectivity*, while *increased long-range connectivity* of the whole thalamus was observed with several cortical sensory areas (Tomasi and Volkow, 2019), correcting previously assumed characteristics of connectivity in ASD.

The anterior thalamus is a brain structure that contains the ventral anterior and the dorsomedial nuclei with their projection to the prefrontal cortex and to primary/association visual, auditory and somatosensory cortical areas (Behrens et al., 2003). With growing age this area showed an increase of local functional connectivity density (lFCD) in both groups, ASD and HC, but significantly less so in ASD (Tomasi and Volkow, 2019). The degree of local connectivity reduction in the anterior thalamus compared to HC was positively associated with symptom severity in ASD (Tomasi and Volkow, 2019). Local connectivity correlates positively with the brain glucose metabolism, which reflects activity state and energy demand of the brain (Tomasi et al., 2013). The whole thalamus showed higher functional connectivity with the insula, somatosensory, motor, premotor and auditory areas and the middle cingulum for ASD compared to HC (Tomasi and Volkow, 2019). These neuroanatomical areas are associated with core symptoms of ASD: social impairment is linked to the *temporal sulcus*, language and communication dysfunction to the *thalamus/superior temporal sulcus/premotor cortex* and repetitive, stereotyped behavior to the *thalamus* and *motor areas* of the cortex amongst others (see Amaral et al., 2008).

Hence, the thalamus seems to be a key region for understanding ASD neuropathology given no other brain region with significant findings of connectivity abnormalities between HC and ASD patients was found in this large sample (Tomasi and Volkow, 2019). On the one hand the *thalamus* is mainly responsible for filtering information for regulated consciousness and alertness. It also integrates sensory and motor signals (Bell and Shine, 2016). Simplified, what passes through the thalamus comes to our awareness. The anterior thalamus with its observed local under-connectivity (Tomasi and Volkow, 2019), leads us to the assumption that in this region, that assesses sensory information of different qualities with respect to their importance of transmission, local communication and activity between neurons is impaired or disrupted. There is no clear evidence whether only the excitatory or inhibitory system or even both are affected in the anterior thalamus due to the macroscopic methods used (Tomasi and Volkow, 2019). It would be plausible though that both systems are affected in a quite individual way.

Many details of sensory information might pass through this physiological filter, with the whole thalamus showing increased projections to several brain areas. Increased long-range connectivity to different sensory areas might be an explanation for sensory abnormalities in ASD, such as hypersensitivity or sensory overload (O’Neill and Jones, 1997; Bromley et al., 2004; Harrison and Hare, 2004). In keeping with this line of thought is the report of abnormal resting states in EEG in ASD (Wang et al., 2013) that might be caused by the increase of long-range connectivity and could explain the *signaling imbalance theory* relating to elevated excitation and reduction of inhibition in brains of people with ASD, as well as the association of ASD with epilepsy (Croen et al., 2015), which is defined as a disorder of neuronal hyper-excitation. Furthermore, long range overconnectivity of the thalamus could account for autonomous nervous system (ANS) dysfunction in ASD (Panju et al., 2015). ANS dysfunction is proposed to be related to sympathetic hyper-arousal and a lower parasympathetic tone, shown by an increased heart rate, larger tonic pupil size and decreased heart rate variability (HRV) (Bal et al., 2010; Daluwatte et al., 2013; Porges et al., 2013; Kushki et al., 2014; Panju et al., 2015), what can be seen as symptoms of distress.

In fact, neuronal hyper-excitation and the associated chronic distress is in line with a whole series of findings of somatic complications found increased in ASD. For instance, increased neuronal activation in the CNS and ANS due to dysfunctional abnormalities of thalamocortical connectivity might explain why sleeping disorders are commonly found in ASD (Aldinger et al., 2015). Sleep is highly controlled by the circadian clock system where adaptation to the surrounding environment, like day and night, is fundamental. Associated with sleep disorders are gastrointestinal disturbances (GID), which are likewise commonly found in ASD (Klukowski et al., 2015). Children with ASD are often affected with autoimmune disorders, allergies, GI disorders, sleep disorders and seizures (Croen et al., 2015), while adults with ASD often suffer from chronic medical conditions, including dyslipidemia, hypertension, diabetes, obesity and thyroid disease (Croen et al., 2015). Especially the prevalence of stroke and Parkinson’s disease, as well as vitamin deficiency is also significantly increased in individuals with ASD (Croen et al., 2015).

### The Circadian Clock in ASD

Hypothesis 2: Abnormal neuronal connectivity via altered excitation (glutamate)/inhibition (GABA) leads to dysregulated melatonin synthesis affecting the circadian rhythm and genetic transcription.

**FIGURE 1:**
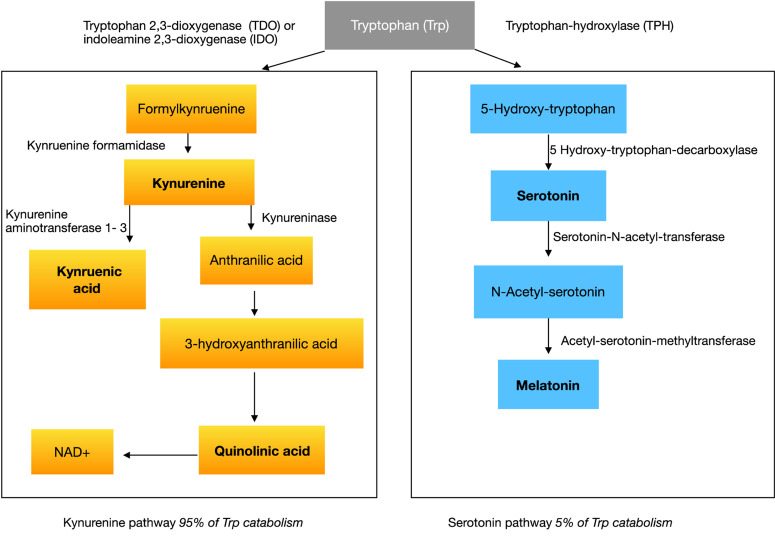
Tryptophan metabolism.

With respect to the large prevalence of sleep disorders around 50–80% (Richdale and Schreck, 2009; Souders et al., 2009; Mazzone et al., 2018) in ASD, several studies investigated melatonin or melatonin metabolites showing abnormalities in ASD (Rossignol and Frye, 2011). Melatonin is an endogenous neurohormone transmitted mostly by the pineal gland, synthesized from serotonin in a two-step pathway. The common amino acid synthesized into serotonin and melatonin is tryptophan (see Figure 1).

Melatonin is important for the circadian clock in mammals. Beside its function in regulation and adjustment to exogenous stimulation, by day and night, it has an important role as antioxidant. Its immunomodulatory function is unclear, but there is the concept of melatonin as an “immune buffer” that has an anti-inflammatory compound during acute inflammation (Carrillo-Vico et al., 2013). On the assumption of the immunosuppressive role of melatonin and its decreased concentration in ASD, many individuals with ASD should show abnormalities in their immune system and its peripheral immune cell concentrations (Rossignol and Frye, 2012; Malkova and Hsiao, 2016), as well as gastrointestinal inflammatory diseases. Indeed, all of these are known comorbidities highly prevalent in ASD (Croen et al., 2015).

The key brain region associated with melatonin is the nucleus suprachiasmatic nucleus (SCN) that gets activated by different pathways of the visual system. Synthesis and release of melatonin in the pineal gland and retina follows a circadian rhythm. The SCN or melatonin itself is regulating many other circadian clock dependent systems, such as temperature, blood volume, behavior, locomotor activity, water balance, metabolic and immune functions (Bass and Takahashi, 2010; Mohawk et al., 2012; Scheiermann et al., 2013; Curtis et al., 2014; Labrecque and Cermakian, 2015). The aim of the body circadian clock is to synchronize rhythms and gene expression to a constantly changing environment in order to save homoeostasis in the whole organism. In order to achieve this kind of inner balance and efficient cellular responses, synthesis of melatonin and its binding to receptors, as well as receptor sensitivity, needs to be well regulated (Dubocovich et al., 2003).

Regulating factors are substances such as vasoactive intestinal peptide (VIP), neuropeptide Y, opioids, GABA, dopamine and glutamate (Dubocovich et al., 2003). If glutamate as an excitatory neurotransmitter and GABA as an inhibitory molecule are in an abnormal relation to each other, as mentioned in the *signaling imbalance theory* of autism, regulation of melatonin synthesis gets affected. Glutamate is an important modulatory molecule that inhibits melatonin synthesis by decreasing its genetic expression and the activity of its key enzyme (Villela et al., 2013). High glutamate levels might be one factor amongst others causing lower melatonin concentrations in ASD (Rossignol and Frye, 2011).

Melatonin is also meant for regulating synaptic plasticity (Frank, 2016). Periodic waves of the GABAergic inhibition in the hippocampal circuits are provided by the SCN (Frank, 2016). Dopaminergic synapses of the striatum show plasticity, while dopamine synthesis and metabolism is following a rhythmic expression due to the direct transcriptional link of dopamine gene activation by the core clock (McClung, 2007; Parekh et al., 2015).

Findings that melatonin production in adolescents and young adults with ASD is lower compared to HC (Tordjman et al., 2012) leads to the assumption that the circadian clock system is also affected in autism. Dysregulation of the circadian clock system and its clock genes might be caused (Nicholas et al., 2007, 2008) not mainly from genetic mutations. A lack of resting and sleep disturbs the normal shaping process of synaptic connections (Frank and Cantera, 2014). If the inner-circadian clock system gets disrupted, gene transcription and translation is necessarily affected negatively. For instance, abnormally low melatonin concentrations in ASD intensify sleep disorders and abnormal synaptic plasticity in the brain via dysregulation of neurotransmitters. This effect is bidirectional and can reinforce the impairment of the circadian clock system. Sleep quality affects the ANS and the immune system. Studies about the circadian clock function and its effects on our behavior describe the phenomenon of jet-lag, a misalignment of internal circadian rhythms and external time (Comperatore and Krueger, 1990; Waterhouse, 1999). Symptoms resulting from jet-lag are insomnia (Arendt, 2009), decreased alertness and impaired cognitive skills. Chronic jet-lag is supposed to cause depressed mood, reduced psychomotor coordination and gastrointestinal disturbances (Waterhouse et al., 2007). Many of these symptoms also fit the comorbidities found in ASD. Chronic jet-lag in rodents was shown to lead to an increased risk of cardiomyopathies (Penev et al., 1998) and early death (Davidson et al., 2006), risks that are also found to be markedly increased in individuals with ASD (Croen et al., 2015).

The SCN synchronizes peripheral oscillators in several organs via hormonal and neuronal pathways. In one organ different subgroups of clock genes exist distinguishable on the basis of their transcription rate and velocity. Different periodic rhythms exist directly besides each other controlled by the main pacemaker, the SCN, and the hormone melatonin. Therefore, temporal disorganization of the circadian system during jet-lag likely disrupts overall physiological coordination. Indeed, reduction of melatonin-functioning was found to correlate with the severity of ASD symptoms (Rossignol and Frye, 2011).

### The Immune System in ASD

Hypothesis 3: Chronic distress, sleep disturbance and disruptions of the circadian clock system and melatonin homeostasis result in increased cortisol concentration and immune system disarrangement. Due to the assumption of a bidirectional pathway, we understand this as a self-reinforcing process in ASD.

Disruption of the circadian clock system and sleep plays a critical role in immune system homeostasis (Castanon-Cervantes et al., 2010). Innate and adaptive immune responses are regulated in a time of day-dependent manner (Haspel et al., 2020) Melatonin, a potent antioxidant, is known to have pleiotropic effects on the immune system (Carrillo-Vico et al., 2013). Glutamate’s inhibitory effect on melatonin synthesis involves interactions between astrocytes and pinealocytes, through the release of astrocytic TNF-alpha, a potent mediator of inflammation (Villela et al., 2013). TNF-alpha, a proinflammatory molecule, stimulates amongst others the release of corticotropin-releasing hormone (CRH) from the hypothalamus (Watanobe and Takebe, 1992). CRH activates via ACTH the secretion of glucocorticoids, like cortisol. High levels of neuronal glutamate might therefore not only decrease melatonin levels in the SCN, moreover it elevates inflammatory molecules via paracrine interaction with astrocytes and elevates immune system activity.

Typically, cortisol as an inflammatory corticosteroid hormone gets upregulated in stressful times to protect the body. Other inflammatory markers such as C-reactive protein (hs-CRP), cytochrome P450 (CYPp450) and 8-hydroxy-2′-deoxyguanosine (8-OH-dG) are blood plasma biomarkers related to inflammation and oxidative stress that were shown to be increased in ASD (Rossignol et al., 2014). Indeed, a higher prevalence of immune dysfunction is found in children with ASD (Croen et al., 2015). Moreover, the disruption of the circadian clock has an effect on the immune system as well because of its regulation of circadian clock genes in the adrenal gland where glucocorticoids, such as the hormone cortisol, are secreted.

Chronic distress is caused physiologically by the reaction of the HPA-axis and the ANS. Their hyperactivity can lead to several other disorders (Mc Ewen, 2006). For instance, a pathological HPA-axis functions as a predictor for cardiovascular diseases as well as for type 2 diabetes (Rosmond and Björntorp, 2000). Both are two somatic comorbidities significantly increased in autistic individuals (Croen et al., 2015). Of course, this dysregulation pathway is not characteristic or specific for ASD but it is quite important to mention, given melatonin being an antagonist of cortisol. In general, if melatonin is low in ASD patients, as shown above, cortisol gets upregulated. Several findings support the concept of abnormalities in stress response in ASD also at the cellular level (Essa et al., 2013; Rossignol et al., 2014). Reduced antioxidant defense is reported in several neurological diseases (Essa et al., 2013). There is high evidence that an increase of oxidative stress has also an impact on the pathology of ASD (Essa et al., 2013). Markers of oxidative stress correlate with ASD severity (Rossignol et al., 2014). Moreover, there is the assumption that observed oxidative stress is a chronic condition in autistic individuals (Rossignol et al., 2014). Several studies have reported an elevated production of oxidative markers, an increased exposure to environmental pro-oxidants and a decrease of antioxidant in ASD (Essa et al., 2013). Abnormally low antioxidant levels index a low functioning oxidative stress response. A significant increase of an oxidative stress marker, lipofuszin, is reported in three language areas of autistic people compared to controls (López-Hurtado and Prieto, 2008), while other studies were able to show higher immunoreactivity in several brain areas of ASD individuals (Rossignol et al., 2014). These findings lead to a higher secretion of free radicals with their potential to damage various structures of human brain and to influence CNS development negatively. Oxidative stress is not only interesting in times of brain development, rather it is a factor with impact on cell and membrane integrity, excitotoxicity and energy metabolism (Essa et al., 2013), dynamically in interaction with environmental factors and molecules of the immune system.

The immune system, as a link between genes and environment, is assumed to be affected in ASD (Ashwood and Wakefield, 2006; Rossignol and Frye, 2012). Therefore, due to the individual amount of severity of neurological, somatic and genetic abnormalities, flexible adaptation to the environment and an adequate stress response down to the cellular level, is hampered, with impact on a cognitive and psychological level. This is congruent with an understanding of autism as a condition of generalized imbalance in adaption.

### Social Functioning and Oxytocin in ASD

Hypothesis 4: Higher levels of stress, with a low functioning oxidative stress response and elevated cortisol concentration in ASD cause downregulation of oxytocin secretion and gene expression and increased methylation of the OXTR gene, overall with impact on social behavior and interaction.

Social interaction is often reported as being stressful for autistic people (Corbett et al., 2010). The physiological correlate for stress is the activity of the HPA axis and its secretion of ACTH and cortisol.

Several studies have investigated cortisol levels and its circadian rhythm in autistic individuals. An elevation of fetal cortisol concentration has been reported (Baron-Cohen et al., 2015) with potential impact on early CNS development. Moreover, there is evidence that children with autism show a more variable cortisol rhythm and a significant elevation of cortisol following exposure to a novel, non-social stimulus (Corbett et al., 2006). Further investigations found a higher serum cortisol response, with significantly higher peak cortisol levels and prolonged duration and recovery of cortisol elevation following a stressor in ASD (Spratt et al., 2012). Cortisol levels during a peer-interaction task and after the game differed significantly between ASD and TD children, with higher levels in the ASD group (Corbett et al., 2016). Higher physiological arousal during playing was associated with heightened sensory sensitivity and increased stress in autistic children (Corbett et al., 2016). These findings lead to the assumption of an increased reactivity of the HPA axis to stress and novel stimuli in autism with a higher cortisol level measured peripherally.

Furthermore, cortisol is important in understanding the physiological role of oxytocin. Oxytocin is meant to modulate the stress response, by regulating cortisol and cytokine concentration inversely (McQuaid et al., 2016). Given that central oxytocin administration reduces stress-induced corticosterone release and anxiety behavior (Windle et al., 1997), leads to the conceptualization of an existing antagonism between the concentrations of oxytocin and cortisol in the CNS. Much research is done to study the question of correlation of autistic behavior and dysregulated oxytocin concentration (Modahl et al., 1998; Al-Ayadhi, 2005). Oxytocin is a neuropeptide produced in the hypothalamus and released by the posterior pituitary gland that plays a role in social bonding, childbirth and sexual reproduction. Social bonding is impaired, while the prevalence of anxiety is increased (Croen et al., 2015) in individuals with ASD suggesting less sensitivity to oxytocin caused by an abnormality in oxytocin receptor (OXTR) density during an early life period (Freeman et al., 2018). Accordingly, increased OXTR methylation in specific promoter regions (Gregory et al., 2009), as an effect of epigenetics, is in line with lower expression of the OXTR (Kusui et al., 2001) in ASD.

Thus, on the basis of a cascade of reduced local inhibition and increased long-range connectivity, with an assumed early-lifetime impact on the developing brain, leading to hyper-activity in the cerebral cortex and increased levels of cortisol, the proposed framework offers an account of lowered oxytocin as indeed observed in ASD (Modahl et al., 1998). This does not rule out an additional genetic coding dysfunction for oxytocin as well as for glucocorticoids (Brkanac et al., 2008; Patel et al., 2016).

### Neuroendocrinology in ASD

Hypothesis 5: Higher inflammatory signaling molecules such as glucocorticoids in ASD shift the ratio from reaction pathways of tryptophan in favor of kynurenine rather than serotonin. This leads to another imbalance with the result of too much kynurenine, low tryptophan and serotonin as well as, referring to further reactions, low melatonin.

The synthesis pathway of melatonin leads us to the amino acid tryptophan, which is essentially converted to serotonin in the first step of melatonin synthesis (see Figure 1). If there would be a lack of serotonin or tryptophan in the brain in the first instance, then not enough substrate would be available for further reactions to melatonin in the pineal gland.

Tryptophan is an essential amino acid, which must be supplied in the diet (Le Floc’h et al., 2011), usually representing a component of protein. Once absorbed from the gut it can exist free or albumin-bound in circulation. Tryptophan can cross the blood-brain-barrier (BBB) and takes part in the synthesis of serotonin in the central nervous system (CNS). There is evidence that individuals with ASD have low tryptophan concentrations peripherally (Kałużna-Czaplińska et al., 2017). Serotonin itself cannot cross the BBB, even though more than 90 percent is located in enterochromaffin (EC) cells of the gastrointestinal tract (Gershon and Tack, 2007). A lack of central tryptophan would lead to less serotonin as well as lower melatonin concentration in the brain.

Tryptophan is converted in a first step to 5-hydroxytryptophan (5-HTP) by the rate-limiting enzyme, tryptophan hydroxylase (TPH) (see Figure 1). Two isoforms of this enzyme exist, TPH1 and TPH2. They are both in different kinds responsible for the serotonin-synthesis in the enteric nervous system (ENS) and CNS. In the second step 5-HTP is converted to serotonin. Tryptophan gets dominantly transformed by the kynurenine pathway. Kynurenine is produced from tryptophan by two different enzymes: tryptophan 2,3-dioxygenase (TDO) and indoleamine 2,3-dioxygenase (IDO) (see Figure 1). TDO can be induced by glucocorticoids or indeed tryptophan itself. IDO is affected by certain inflammatory stimuli, such as IFN-gamma.

Hypothesis 6: We propose that a lower concentration of kynurenic acid and a higher concentration of QUIN via the increase of oxidative stress and the release of glutamate aggravates the imbalance of the ratio of excitation/inhibition in the brain and has a neurotoxic effect.

Kynurenine itself is metabolized along two distinct pathways. The first one leads to the production of the neuroprotective kynurenic acid (a7 nicotinic acetylcholine receptor antagonist and N-methyl-d-aspartate (NMDA) receptor antagonist at glycine site) while the second arm leads to the neurotoxic quinolinic acid (NMDA receptor agonist) (see Figure 1).

Peripheral measurements showed an imbalance in homeostasis of the kynurenine pathway products with higher levels of QUIN in autistic children (Gevi et al., 2016) and lower levels of kynurenic acid (Bryn et al., 2017), while the ratio between kynurenine and kynurenic acid was significantly higher in the ASD group (Bryn et al., 2017). This ratio reflects a neurotoxic potential. Abnormally high concentrations of QUIN in the CNS of individuals with ASD might be also caused by higher levels of its substrate kynurenine. QUIN is a neurotoxic molecule. It increases oxidative stress by elevating the production of free radicals as well as increasing glutamate release and inhibiting its reuptake by astrocytes (Tavares et al., 2002). The latter aspect results in an elevated concentration of glutamate, leading to overstimulation of NMDA receptors, that cause disturbances in intracellular Ca2 + -signaling by weakening the sarco/endoplasmic reticulum Ca2 + ATPase (Fernandes et al., 2008). Elevation of glutamate by QUIN might have the potential to aggravate the excitation:inhibition imbalance in brain.

Consequences on intracellular signal cascades are also in line with alterations of genetics, like calcium and MAPK signaling pathways (Wen et al., 2016). These several influencing factors might intensify abnormalities in intracellular communication by long-term adaptation to this altered intracellular state. Moreover, we assume that altered internal balance on cellular and neurophysiological levels is one of the main reasons leading to a lower ability in ASD to adapt to the environment and own internal changing states.

### The Gut-Brain Axis in ASD

Hypothesis 7: Alterations of microbiome composition in ASD weaken the availability of tryptophan peripherally and cause disturbances in the endocrine balance by maladaptation of feedback loops and generally misbalanced adaptation to the environment.

Brain and gut communicate through the gut-brain axis, where serotonin is meant to be a linking molecule (Fattorusso et al., 2019). The gut microbiota, a complex of bacterial community located in the GI tract, has been found to be essential for maintaining metabolic and immune health (Lynch and Pedersen, 2016). There is even more evidence that the composition of the microbiome influences brain development, neurogenesis and interacts with the ENS and CNS via the gut-brain axis. Bacteria have been found to have the capability to produce a range of major neurotransmitters, also known under the term “*microbial endocrinology*.” Gut microbes are known to regulate the serotonin concentration in the blood and colon (Yano et al., 2015) via their production of short-chain fatty acids (SCFAs). SCFAs can also modulate the activity of the host’s sympathetic nervous system (Kimura et al., 2011). By using a variety of preclinical strategies, it has been established that manipulating the composition of the gut microbiota across the lifespan or altering the trajectory of microbial colonization of the gastrointestinal tract quite early in lifetime influences the availability of tryptophan (O’Mahony et al., 2015). Interestingly, animal studies have shown that early life time distress leads, beside the observed dysbiosis in the microbiome, also to an increase of immune system and HPA axis activity (O’Mahony et al., 2009). These alterations are meant to persist over lifetime and have adverse effects on behavior, such as on regulation of the stress neurocircuitry, emotions and cognition.

Changes in the composition of the microbiome, called “*microbial dysbiosis*,” have been reported in ASD (Van Sadelhoff et al., 2019). We assume that this alteration of composition is linked to an individually reduced availability of tryptophan in general, resulting in low tryptophan levels in ASD (Kałużna-Czaplińska et al., 2017). The reduced availability of tryptophan increases the activation of the sympathetic nervous system by SCFAs, consistent with our assumption of increased ANS and HPA activity. And consequently, the serotonin synthesis is upregulated peripherally in EC cells via bacterial metabolites, congruent to observed elevated serotonin concentrations peripherally in ASD, called hyperserotonemia (Hranilovic et al., 2007). Thus, we need to assume weakened feedback loops of tryptophan metabolism in ASD on the basis of an altered microbiome composition.

Hypothesis 8: Impaired genetic signaling pathways reduce intestinal epithelium barrier integrity aggravating proinflammatory state in ASD.

Moreover, cellular signaling cascades, like the WNT pathway, do exist not just in the CNS. WNT signaling is also important as a regulator in the intestinal mucosa by organizing epithelial stem cell identity and maintenance (Moparthi and Koch, 2019). Mutations of genes of the canonical WNT pathway might therefore result in lower intestinal epithelium integrity (Pinto et al., 2003). As a consequence, pathogens of the daily environment and metabolism have the opportunity to enter cells more easily and harm the host more effectively, leading to an increase of proinflammatory molecules by upregulation of the host’s immune system and HPA-axis activity for defense.

Hypothesis 9: Alterations of microbiome composition in ASD aggravate the dysregulation of the tryptophan metabolism by increasing kynurenine levels peripherally and centrally, in line with weakened feedback loops in ASD.

Central effects of serotonin are related to the circadian rhythm, motor control, body temperature, vascular tone and cerebellar regulation, while in the gastrointestinal system serotonin regulates pancreatic, intestinal and gastric secretion, gastrointestinal motility and colonic tone. While tryptophan can cross the BBB, its availability is necessary for the amount of serotonin in the brain. Several gut bacteria can modulate the metabolism of tryptophan into kynurenine. Depending on the bacteria involved, kynurenine biosynthesis can be increased or decreased. Some probiotics have been shown to reduce kynurenine levels (Desbonnet et al., 2008), for instance. Given the microbial dysbiosis in ASD we assume that this alteration in the gut increases the concentration of kynurenine peripherally via the tryptophan metabolism and in the CNS via the BBB. Hence, the peripheral shift of the tryptophan metabolism toward more kynurenine and reduced serotonin is mirrored in the CNS (see Hypothesis 5).

Furthermore, through an increase of the proinflammatory state in the gut (see Hypothesis 8) the enzymes of the kynurenine pathway get upregulated, another self-reinforcing process in the periphery.

Finally, elevated kynurenine levels lead us back to the neurotoxic effect of QUIN and the decreased neuroprotective potential of kynurenic acid (see section “Neuroendocrinology in ASD”), further aggravating the previously explicated effect of an activity increase of ANS/HPA via altered connectivity in ASD (see section “Connectivity in ASD”), on the basis of genetic (see section “Alterations of Neurodevelopmental Signaling Pathways in ASD”) and environmental modulators (see sections “The Circadian Clock in ASD” and “The Gut-Brain Axis in ASD”).

### The Model – The Generalized Adaptation Framework of Autism

**FIGURE 2:**
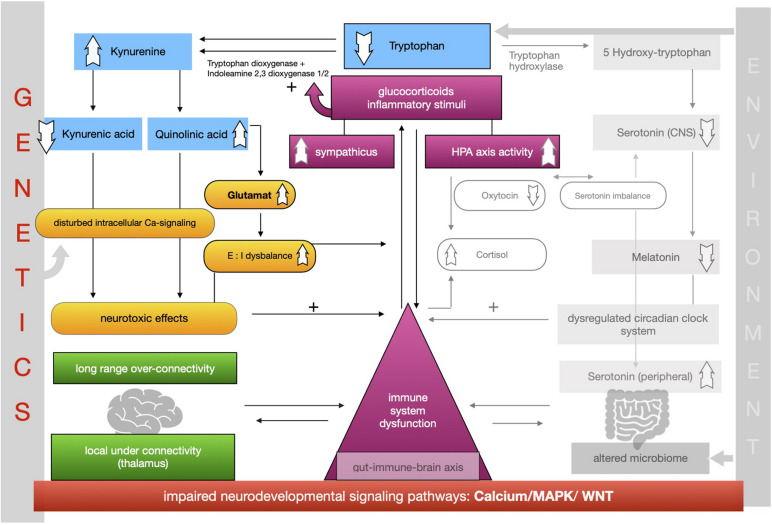
Feedback circuit A.

**FIGURE 3:**
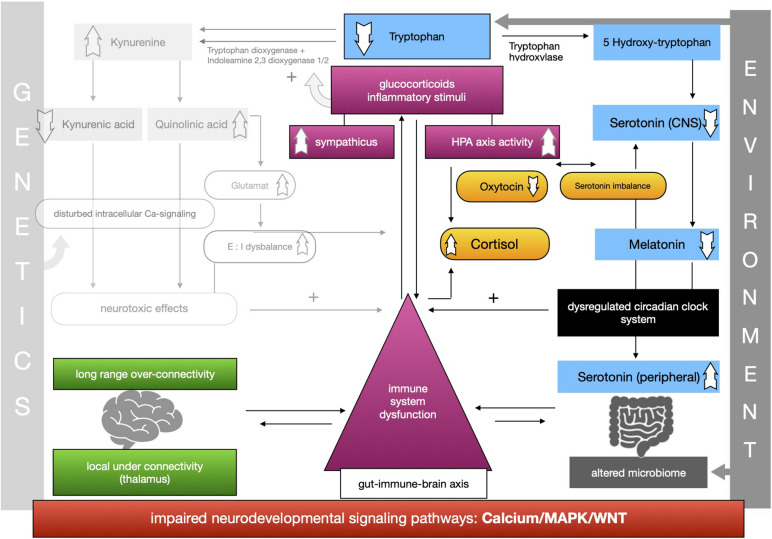
Feedback circuit B.

The proposed framework of ASD as a condition of generalized imbalance in adaptation can be subdivided into two intertwined negative feedback circuits (see Figures 2, 3, respectively) under the umbrella of genetic alterations and the environment. Both feedback circuits are highly variable between individuals in line with the quite heterogeneous spectrum of ASD.

Following the first circuit (see Figure 2), WNT, calcium and MAPK signaling pathways are negatively affected in ASD and epigenetically relate to gene × environment interactions. Abnormal signaling cascades lead to alterations in the formation of synapses, intracellular communication and the excitation/inhibition ratio as well as to increased levels of neuroligins. On this basis we think that the findings of long-range overconnectivity and local underconnectivity of the thalamus in ASD lead to a lower filter function of information in the brain. The resulting simultaneous activity of different cortical areas causes higher activity of the ANS and HPA-axis and consequently increased secretion of proinflammatory molecules and glucocorticoids. These molecules intensify the kynurenine pathway of the metabolism of tryptophan (see Figure 2). Kynurenine thereby gets upregulated in its concentration leading to higher concentrations of QUIN in the brain. Kynurenic acid is lower and in relation to kynurenine it has a neurotoxic effect. QUIN increases the release of glutamate in the CNS in line with an imbalance of excitatory and inhibitory neurons. The neurotoxicity might also result in graded levels of cognitive impairments. Importantly, it also results in a reinforcing process by activation of the immune system for defense. Higher concentrations of proinflammatory stimuli could raise the level of kynurenine as a positive modulator of the two enzymes tryptophan-dioxygenase and indoleamine-2,3-dioxygenase. We assume that mismatched feedback loops are existent in this circuit.

The second circuit (see Figure 3) again starts off from altered signaling pathways and connectivity in ASD with higher HPA-axis activity and stress levels (see Figure 3). As mentioned, we propose that the kynurenine pathway is upregulated by response to higher stress levels in ASD. This results in a shift of the balance of the two possible reaction pathways of tryptophan and lower serotonin and melatonin concentration in the CNS. Lower melatonin leads to disturbances in the circadian clock system causing sleep disorders and higher distress and enhancing the activity of the immune system. The imbalance of excitatory/inhibitory neurons, as presented in the first circuit, aggravates the dysregulation in melatonin synthesis via their regulatory effects. The circadian clock system stands as an example for the impaired adaptation ability to the environment in ASD. The relationship between melatonin and cortisol, a typical stress hormone, is antagonistic. Here too there is a reinforcing process. The HPA-axis hormone oxytocin is antagonistically reduced, which is in line with reported impairments of social bonding and interaction in ASD. Meanwhile, the environment impacts the intestinal system where alterations of the composition of the microbiome are commonly observed in ASD. Due to altered signaling pathways, we assume that intestinal and BBB integrity is weakened so that more pathogens and other toxic substances can enter cells more easily and reinforce the activity of the immune system. In this second circuit (Figure 3) we likewise propose weakened feedback loops leading to maladaptation of inner cellular and hormone pathways, especially in the gut-brain axis and its linking molecule serotonin. Microbial alterations lead to lower levels of tryptophan peripherally, while serotonin concentration is increased due to the stimulation of EC cells by SCFAs, a product of bacterial metabolism. Moreover the microbial dysbiosis in ASD might strengthen the imbalance of tryptophan metabolism in the CNS and gut by increasing the levels of neurotoxic kynurenine, leading to decreased serotonin as well as tryptophan concentrations in the brain. Higher activity of the immune system caused by the decreased epithelium barrier integrity reinforces the kynurenine pathway by enzyme stimulation.

Both circuits are intertwined into one whole self-reinforcing process in ASD, which is the basis of a generalized impairment of adaptation to the environment and one’s own internal states. A comprehensible way to adapt to the lack of homeostasis is stereotypical and repetitive behavior, as an early learned regulatory self-stimulation that helps people with ASD in situations that are experienced as stressful due to the fact that this kind of behavior requires less adaptability.

**FIGURE 4:**
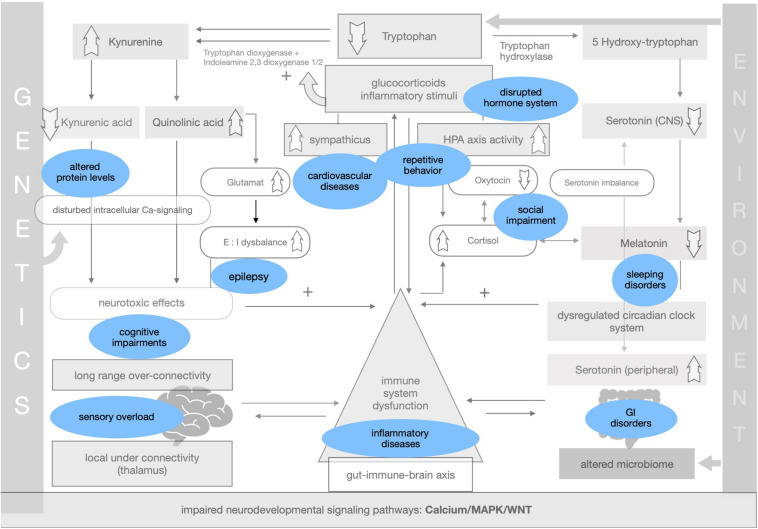
Symptoms of ASD in the generalized adaptation framework.

Symptoms and comorbidities linked to ASD can be implemented within the theory of impaired adaptation (see Figure 4).

**Box 1. Outlook on potential treatment options in the generalized adaptation framework.**

One suggested treatment plan would be to focus more on nutrition, especially in the subgroup of autistic children, to avoid obesity and associated medical comorbidities like cardiovascular and metabolic diseases. Constant blood screening of autistic people for inflammatory biomarkers might be helpful to see whether they are elevated or not and how long inflammation takes. Even more there should be also the focus on screening levels of corticosteroids, like cortisol and its metabolites, due to their suggested antagonistic effect with melatonin and oxytocin.

Drug treatment in general should carefully consider co-occurring effects on the circadian clock and the sympathetic nervous system. Based on the thesis of neuronal hyperactivity the aim should be to reduce the level of activation of the sympathetic nervous system relatively in ratio to the parasympathetic one in order to care for good sleep, lower stress level, lower concentrations of inflammatory markers as well as for lower risk suffering from epileptic attacks and cardiovascular disorders. Relieving the HPA-axis and the ANS via the sympathetic pathway might be an effective treatment in future times for ASD symptoms.

A possible drug for sleeping disorders could be melatonin, especially in childhood. Several studies could show quite good evidence for improved sleep parameters and better daytime behavior (Rossignol and Frye, 2011). The focus lays on the attempt to resynchronize the circadian clock to environmental stimuli so that gene transcription and translation work more regularly and that regulation of all several subgroups of clock genes in the different tissues are not disturbed that much as without melatonin treatment because of the primary pacemaker function of melatonin in the chronobiology system.

Propranolol, a non-selective beta-blocker, is inhibiting the noradrenaline and adrenaline system. After oral administration, it gets absorbed up to 90% in the liver. Usually it is used for treatment in hypertension and angina as well as migraine. Contraindications are bronchial asthma and bronchospasm because of increasing these symptoms. Propranolol is lipophilic and enters the BBB, so that it gets used for treating anxiety disorders. Propranolol reduces autonomic dysregulation by blocking the sympathetic nervous system. Therefore, it can be useful for treating disorders concerning to emotional and behavioral deficits caused by hyperarousal. Sixteen reports are found in a review about the use of propranolol in ASD (Sagar-Ouriaghli et al., 2018). The results from the eight single-dose clinical trials led to significant improvements in cognitive performance, improvement in semantic networks and functional connectivity. The remaining eight single case reports and case series showed improvements in anxiety, aggressive, self-injurious and hypersexual behaviors. In no study a negative observation has been reported so far for the treatment with this kind of beta-blocker apart from high dose treatment that caused hypotension (Sagar-Ouriaghli et al., 2018). This can be seen as a treatable side effect. It should be mentioned that autistic individuals with high dysregulation in the autonomous nervous systems and low functional connectivity gained the greatest benefit from propranolol treatment.

Further research should be done beside the use in clinical practice of propranolol, whether there are other lipophilic beta-blocker molecules that have a similar effect and are suitable for ASD treatment.

### The Specificity of Maladaptation in ASD

While all neuropsychiatric conditions might have elements of maladaptation to the environment (e.g., sleep disorders are widely associated with several neuropsychiatric conditions), there are two important characteristics differentiating maladaptation in ASD from that potentially to be found in other neuropsychiatric conditions. First, deviant adaptation to the environment in ASD is arguably present from birth, given the neurodevelopmental nature of ASD, in contrast to late acquisition of potential deviant adaptation in neuropsychiatric conditions, such as depression. Maladaptation during early neurologically vulnerable phases of development would arguably strongly shape individual developmental pathways. According to the neuroconstructivist perspective (Karmiloff-Smith, 2006) we need to take the ontogenetic development into account that is continuously forming the microconnectivity of the brain and the fine-tuning of functional circuits. Importantly, the neurodevelopmental perspective with cascading effects of constricted adaptation throughout levels of functioning *per se* entails the generalized nature of deviant adaptation.

Second, deviant adaptation to the environment in ASD would differ from that potentially found in other neuro*developmental* disorders, in that in the latter case it would be confined to specific aspects of brain development and neurological functioning. For instance, although adaptation problems are clearly observable in Attention-Deficit/Hyperactivity Disorder (ADHD), these are primarily confined to executive functioning. In contrast, in ASD deviant adaptation is thought of as a pervasive process in that it is thought to affect states of metabolism, neuronal connectivity, cognition, immune system, social interaction, and individual somatic levels. This neurodevelopmental pattern of pervasive deviant adaptation combined with the incapacity of a physiological transformation process during development is a specific pattern within ASD.

Thus, the presented framework points toward the importance of environmental factors to be adapted to each individuals’ needs and symptom severity to reduce negative somatic effects. In addition, compensatory strategies have to be learnt, and this learning should be supported by tailored interventions, to cope with challenging situations and thereby improve health and life expectancy of autistic people in general.

## Conclusion

The proposed framework seeks to unify most recent findings on neurobiological, endocrinological, cellular and connectivity levels in order to explain association with and gradation of various symptoms of ASD and its comorbidities. The presented model accounts for the phenomenological heterogeneity of the spectrum. The feedback circuits provide the opportunity to alleviate stress reactions, the activity of the immune system and consequently the risk of comorbidities by taking care of dynamical changing environmental factors in each individual case. The theory has the potential to give an explanation why there are also autistic individuals with mild symptoms and a lower risk for comorbidities in line with higher lifetime quality. Concerning repetitive behavior as a possible compensatory strategy in ASD to deal with these several imbalances, the model also highlights strengths of autistic people. The multivariable conceptualization of ASD in the proposed framework as a generalized adaptation imbalance declares, why no one specific key treatment for autistic symptoms can be established. While deviant adaptation is not specific to ASD, the pattern of pervasive deviant adaptation on all the levels described in the framework is argued to be specifically characteristic for ASD - a theoretical framework which should be subject to targeted future research (see Box 1 as an example for future research topics).

## Author Contributions

CG conceived the theory. All authors critically discussed the results and contributed to the final manuscript.

## Conflict of Interest

The authors declare that the research was conducted in the absence of any commercial or financial relationships that could be construed as a potential conflict of interest.
